# Efficacy of Nonpreserved Sodium Hyaluronate Artificial Tears in Dry Eye Disease Patients Treated with Prostaglandin Analogs for Primary Open-Angle Glaucoma: A Prospective, Nonrandomized, Open-Label Pilot Study

**DOI:** 10.1155/2022/1320996

**Published:** 2022-12-02

**Authors:** Giulia Coco, Danilo Iannetta, Igino Febbraro, Ester Elmo, Gianluca Manni

**Affiliations:** ^1^Department of Clinical Sciences and Translational Medicine, University of Rome Tor Vergata, Rome, Italy; ^2^Ophthalmology Unit, DIMES, Alma Mater Studiorum, Ophthalmology Department, University of Bologna, S. Orsola-Malpighi Teaching Hospital, Bologna 40138, Italy; ^3^IRCCS-Fondazione Bietti, Rome, Italy

## Abstract

**Purpose:**

Dry eye disease (DED) can be triggered using preserved ophthalmic formulations or prostaglandin analogs. In this prospective, nonrandomized, open-label pilot study, we evaluated the efficacy of a 0.15% hyaluronic acid (HA) nonpreserved ophthalmic formulation in decreasing DED symptoms in patients with open-angle glaucoma treated with prostaglandin analogs.

**Methods:**

30 patients with DED receiving chronic treatment with prostaglandin analogs for primary open-angle glaucoma or ocular hypertension were administered ophthalmic formulations 3 times daily for 12 weeks. Foreign body sensation, burning, stinging, dryness, pain, frequency of symptoms, Ocular Surface Disease Index (OSDI), conjunctival hyperaemia, corneal fluorescein staining (CFS), tear film break-up time (TBUT), best-corrected visual acuity, Schirmer test results, and 25-item National Eye Institute Visual Function Questionnaire score between the baseline and 4 and 12 weeks were evaluated.

**Results:**

The analysis shows that all primary endpoints improved; in particular, burning sensation and the frequency of symptoms after 4 and 12 weeks of treatment (*p* < 0.001) and dryness and pain after 12 weeks of treatment (*p* < 0.001 and *p*=0.03, respectively) were reduced significantly. Secondary outcomes confirmed the positive results, with a statistically significant change in the OSDI score and CFS between the baseline and 4 (*p*=0.02 and *p* < 0.001, respectively) or 12 weeks (both *p* < 0.001) and TBUT after 4 weeks (*p*=0.01). Conjunctival hyperaemia improved in both eyes in >90% of cases at 12 weeks of treatment.

**Conclusion:**

The present study shows that the ophthalmic formulation containing 0.15% HA has a promising beneficial effect on reducing the signs and symptoms of DED in patients treated with prostaglandin analogs.

## 1. Introduction

The ocular surface consists of a continuous epithelium hydrated by the tear film, which also contains protective antimicrobial factors (e.g., defensins, immunoglobulin A, lactoferrin, and lysozyme). Tear film stability is crucial for maintaining homeostasis of the ocular surface and is ensured in particular by the mucin-rich gel produced by epithelial cells [1]. In addition, goblet cells in the epithelium produce cytokines, epidermal growth factor, and retinoic acid, which together maintain immune tolerance [2]. When these protective mechanisms fail, tear deficiency results in alterations in the tear film and hyperosmolar stress, which lead to increased friction and mechanical irritation of the ocular surface [3–5]. In addition to these phenomena, activation of inflammatory processes further increases ocular discomfort [6–9].

According to the 2017 International Dry Eye Workshop II report, dry eye disease (DED) is a multifactorial condition characterized by increased osmolarity of the tear film and inflammation of the ocular surface [10–12]. The prevalence of DED has a wide range (5%–50%) and is estimated to be higher in women, with a tendency to increase with age [13]. Most of the symptoms associated with DED are nonspecific and common to other ocular diseases and include redness, burning, stinging, foreign body sensation, pruritus, and, in some cases, photophobia [13]. The clinical signs of ocular surface inflammation are a loss of conjunctival goblet cells and corneal epitheliopathy [14]. The course of the disease is persistent and characterized by an episodic pattern of symptoms (flares) [15].

Because of the enormous variability of clinical signs, there is no consensus on the diagnosis of DED; nevertheless, self-reported questionnaires, such as the Ocular Surface Disease Index (OSDI), are commonly used as tools for assessing the severity of the disease. In addition, other clinical tests are used by physicians, including the Schirmer test, tear break-up time (TBUT), corneal and conjunctival staining, and tear osmolarity [16].

Many causes underlie the occurrence of DED. The most common are the use of contact lenses, refractive laser cataract surgery, and the use of topical formulations containing preservatives and prostaglandin (PG) analogs for glaucoma and ocular hypertension [17, 18]. Recent studies suggest that the use of topical formulations containing benzalkonium chloride (BAK) as a preservative may have adverse effects on the ocular surface [19]. BAK is a quaternary ammonium compound used in a variety of formulations [20]; although BAK destabilizes cell membranes, leading to bacterial death, its effect is nonspecific and may also affect mammalian cells, resulting in local side effects that are cumulative and become more severe with repeated exposure [21, 22].

Instead, PG analogs have become the first-line therapy for treating patients with glaucoma due to their efficacy in lowering intraocular pressure (IOP) [18]. While reducing IOP, PG analogs are associated with ocular side effects, such as a prominent feature of ocular irritation associated with dry eye disease and an increase in conjunctival hyperaemia [23]. A recent meta-analysis of glaucoma patients showed that the risk of conjunctival hyperaemia increases in patients treated with PG analogs compared to patients treated with other classes of drugs [18, 24]. Conjunctival hyperaemia is thought to be caused by nitric oxide-mediated vasodilation in the conjunctiva. However, the relationship between PG analogs and ocular surface changes is complicated and remains unclear [18].

To date, treatment options for DED have been based on avoidance of triggering factors, such as cigarette smoking, adverse environments, and others, in conjunction with the use of topical nonpreserved formulations, such as artificial tears and corticosteroids or cyclosporin A-based eye drops [11, 25]. Due to the side effects of chronic use of preserved ophthalmic formulations, the commercial trend is increasingly toward preservative-free eye drops.

Hyaluronic acid (HA) is a linear polymer composed of N-acetyl-glucosamine and glucuronate units. Its use in ophthalmology has been studied since the early 1990s, and HA is known to increase tear film stability by stimulating mucin production [26]. Consistent with this notion, existing studies suggest that HA is able to significantly alleviate the symptoms of DED and reduce ocular inflammation [27, 28]. Based on these findings and aiming to keep on providing the literature with increasing clinical data, the present prospective, nonrandomized, open-label pilot study evaluated the efficacy of the formulation containing 0.15% sodium hyaluronate (as the main component), 0.2% *Echinacea* extract, and amino acids in improving DED symptoms.

## 2. Materials and Methods

### 2.1. Aim of the Study

The aim of this prospective, nonrandomized, open-label pilot study was to evaluate the efficacy of a topical HA-based formulation also containing amino acids and 0.2% *Echinacea* extract (Iridium A Free; Fidia Farmaceutici, Padova, Italy) in improving DED symptoms as an adjunctive treatment in patients with primary open-angle glaucoma or ocular hypertension undergoing treatment with PG analogs. This ophthalmic formulation is specifically designed to protect the corneal epithelium and helps increase the biological defence of the tear film by better stabilizing and preserving its properties. The inclusion criteria were as follows: (1) an age of ≥18 years, (2) a diagnosis of primary open-angle glaucoma or ocular hypertension and current treatment with PG analogs as monotherapy or in fixed combination/association with beta-blockers for ≥6 months before enrolment, (3) DED symptoms defined by an Ocular Surface Disease Index (OSDI) score of ≥13 points, (4) a Schirmer test I result of ≥5 mm to avoid the inclusion of dry eye patients due to decreased tear production, and (5) conjunctival hyperaemia of ≥2. Patients were excluded if they (1) used artificial tear substitutes in 2 weeks before the start of the study, (2) had a history of ocular trauma, (3) had an active ocular surface infection of any type, (4) had an ocular allergy, (5) had undergone ocular surgery within 30 days prior to enrolment, (6) had another concurrent eye disease associated with ocular surface inflammation (e.g., pinguecula, pterygium, or corneal scarring associated with corneal irregularities), or (7) were pregnant or breastfeeding. We also excluded patients with DED linked to a systemic disease or therapeutic used to treat a systemic disease and patients with known hypersensitivity to any of the components of the study eye drops. The study protocol was assessed and approved by the Internal Commission of the Clinic; the research was conducted in accordance with the Helsinki Declaration, and patients provided informed consent.

### 2.2. Treatment and Evaluations

Patients, already undergoing treatment with PG analog therapy, were administered 1 drop of ancillary topical therapy containing HA, amino acids, and *Echinacea* 3 times daily for 12 consecutive weeks. The time points considered were baseline, 4 (±1) weeks, and 12 (±1) weeks. At each visit, as per the TFOS DEWS II Diagnostic Methodology report [29], the parameters evaluated were (1) best-corrected visual acuity (BCVA) using a logMAR chart; (2) IOP by Goldmann applanation tonometry; (3) conjunctival hyperaemia measured on a 4-point scale (0 = none, 1 = mild, 2 = moderate, and 3 = severe); (4) tear film break-up time (TBUT) measured after instillation of 1 drop of fluorescein sodium; specifically, one single drop of balanced salt solution (BSS) was applied at the tip of fluorescein strips (AKti-flu fluorescein strips 1 mg sodium fluorescein in each strip; Aktive S.r.l., Italy) and then instilled into the inferior fornix of the patients' eye; patients were then instructed to blink normally for approximately three times and then to stop blinking while TBUT was measured; (5) corneal fluorescein staining (CFS) measured after TBUT according to the National Eye Institute/Industry (NEI) scoring system; (6) ocular surface symptoms using a 10-point visual analog scale (0–10 points) for foreign body sensation, burning, stinging, dryness, pain, and frequency of symptoms; (7) OSDI score; and (8) vision-related quality of life using the 25-item NEI Visual Function Questionnaire (NEI-VFQ-25). The last parameter was evaluated only at the baseline and at 12 ± 1 weeks. All exams were performed in the same environmental settings to avoid potential DED evaluation bias [30]: thermostat-regulated room, dim room light, maximum slit-lamp illumination, same amount of fluorescein, and patients were all evaluated by the same observer. Questionnaires were administered before clinical tests.

### 2.3. Outcomes

The primary outcome of this study was the change in ocular surface inflammatory symptoms for each item of the visual analog scale (foreign body sensation, burning, stinging, dryness, pain, and frequency of symptoms) after 4 and 12 weeks of treatment. The secondary outcomes were the mean change in OSDI score, VFQ-NEI-25, conjunctival hyperaemia, CFS, TBUT, BCVA, and Schirmer test result between baseline, 4 weeks, and 12 weeks after the start of the study.

### 2.4. Sample Size and Statistical Analysis

A medium clinically relevant effect size equal to −0.50 at 12 weeks for the dryness symptom has been considered primary outcome of this study; all other ocular surface DED symptoms of the visual analog scale, such as foreign body sensation, burning, pain, and frequency of symptoms, were also primary outcomes but were not considered for sample size analysis. A sample size of 27 data pairs achieved a minimum of 80% power to reject the null hypothesis of zero effect size at 12 weeks at a significance level (alpha) of 0.10 using the two-sided paired *t*-test. As a rule of thumb, an anticipated 10% dropout rate has been assumed, and thus, the minimum number of evaluable subjects included in the study was *N* = 30.

To select the most appropriate statistical analysis, a preliminary between-eye correlation analysis was performed for all eye-specific outcomes; depending on the value of the Spearman correlation coefficient and visual inspection of between-eye scatterplots, the outcome data were analysed using the average of values from the right and left eyes, or on a per-eye basis, with the exception of symptom scores (VAS and OSDI) which were considered on a per-patient basis.

Continuous variables were summarized by count, mean, standard deviation, and/or interquartile range values by time and analysed with a repeated measures-mixed model. Categorical variables were instead analysed with repeated measures logistic regression analysis, using a subject identifier as a random effect. Regression parameters (*β*) of univariate and multivariate analyses were tabulated as point estimates along with standard errors and *p* values for comparisons between the follow-up and baseline. Multiplicity adjustment was performed using the Student maximum modulus method. Individual profiles and boxplots were created for each primary outcome and for each domain of the NEI-VFQ-25 quality of life questionnaire. A normality test was performed for all variables using the Shapiro–Wilk test. All tests were two-tailed and considered significant at the 5% level. All analyses were conducted using SAS version 9.4 (SAS Institute, Cary, NC, USA).

## 3. Results

### 3.1. Demographics

Thirty patients were enrolled in this study; of these, 15 (50%) were female, and the mean age at the baseline was 64.2 years (standard deviation, 10.5 years). Participating patients had been treated for an average of 9.1 years with PG analogs or a fixed combination thereof in the form of ophthalmic drugs with preservatives for glaucoma. All patients were diagnosed with primary open-angle glaucoma or ocular hypertension, and all had ocular surface inflammation and DED symptoms, such as foreign body sensation, burning, stinging, dryness, and pain at the baseline (mean OSDI value, 37.7 points). All patients included in the study completed the entire treatment period; demographic and baseline diagnostic and treatment details are reported in Table 1.

### 3.2. Correlation Analysis

The left and right eyes were significantly correlated for all primary and secondary outcomes (*p* < 0.001), with Spearman's correlation coefficients ranging from 0.12–0.75. A per-eye analysis was carried out only for conjunctival hyperaemia and BCVA score changes with respect to the baseline.

### 3.3. Primary Outcomes

**Figure 1:**
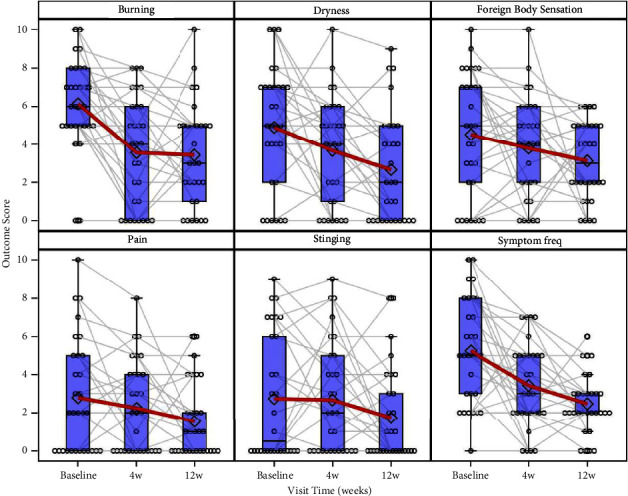
Primary outcomes scores by time with individual profiles.

At both 4 and 12 weeks, all primary outcomes showed a monotonic trend (Figure 1). Treatment resulted in significant improvements in burning and frequency of symptoms at both 4 and 12 weeks. Indeed, the mean values (standard deviation; 95% CI; *p* value) of burning decreased by 2.57 (SD: 2.63; −3.93 to −1.21; *p* < 0.001) and 2.70 (SD: 2.52; −4.00 to −1.40; *p* < 0.001), and the mean values calculated for the frequency of symptoms decreased by 1.80 (SD: 2.51; −3.10 to −0.51; *p*=0.002) and 2.77 (SD: 2.23; −3.92 to −1.61; *p* < 0.001) after 4 and 12 weeks of treatment compared to the baseline, respectively. After 12 weeks of treatment, dryness and pain significantly decreased by 2.23 (SD: 3.02; −3.79 to −0.67; *p* < 0.001) and 1.27 (SD: 2.53; −2.57 to 0.00; *p*=0.03), respectively. We also observed a reduction in foreign body sensation after 12 weeks of treatment; however, the *t*-test yielded a borderline value, and the difference was not statistically significant (mean 1.40; SD: 2.73; −2.81 to 0.00; *p*=0.05). The differences in stinging values at 4 weeks (mean: 0.07; SD: 3.09; −1.66 to 1.53) and 12 weeks (mean: −1.00; SD:2.89; −2.49 to 0.49) compared to the baseline were also not statistically significant (*p*=0.10 and *p*=0.20, respectively).

Age at the baseline was significantly and inversely correlated with burning in both univariate and multivariate analyses (regression coefficient = −0.09; standard error = 0.03; *p*=0.01) as well as the visit time (i.e., *p* < 0.001 for both 4 weeks vs. baseline and 12 weeks vs. baseline). No other factors were significantly associated with the observed changes in the primary outcome.

### 3.4. Secondary Outcomes

**Figure 2:**
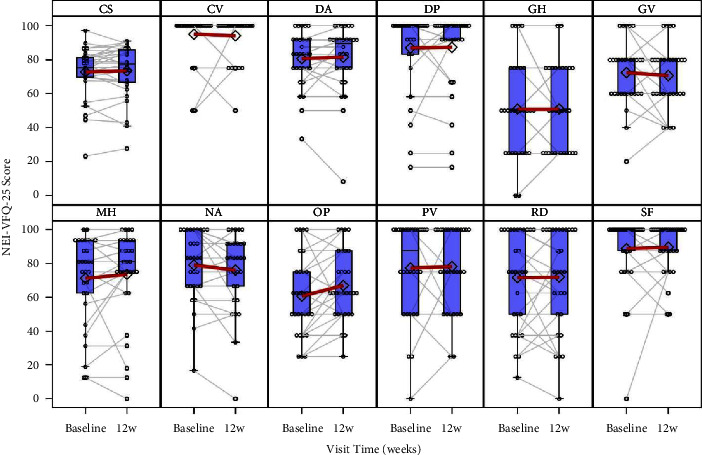
NEI-VFQ-25 domain scores by time with individual profiles. Abbreviations: CS, composite score; CV, color vision; DA, distance activity; DP, dependency; GH, general health; GV, general vision; MH, mental health; NA, near activity; OP, ocular pain; PV, peripheral vision; RD, role difficulty; SF, social functioning.

The OSDI score showed a monotonic and significant mean change between the baseline and 4 and 12 weeks. The mean change scores were −7.8 points (*p*=0.02) and −8.1 points (*p*=0.04) at 4 and 12 weeks, respectively (Table 2). Interestingly, TBUT increased significantly (*p*=0.01) by 1.5 seconds at 4 weeks of treatment compared to the baseline (Table 2). A monotonic and significant decrease in CFS values by 2.1 at 4 weeks (*p* < 0.001) and 4.5 at 12 weeks (*p* < 0.001), respectively, was observed compared to the baseline (Table 2). The results of the Schirmer test showed an increase from the baseline to 12 weeks (from 13.6 to 15.7 mm), which was not clearly statistically significant (*p*=0.05) (Table 2). Due to their distributional properties, a per-eye analysis was performed for conjunctival hyperaemia and BCVA changes at the baseline, with results categorized as worsening, no change, or improvement. Conjunctival hyperaemia improved in both eyes at 12 weeks of treatment in >90% of cases, while BCVA did not change from the baseline for most patients. Indeed, BCVA improvement was observed in the left eye of only 1 (3.3%) patient and the right eyes of 2 (6.7%) patients at both 4 and 12 weeks, and BCVA was worse in the right eye of 1 patient (3.3%) at 4 and 12 weeks. The composite visual quality of life score (NEI-VFQ-25) and corresponding subscales showed no significant changes, except for a decrease in eye pain between the baseline (60.8 points) and 12 weeks (67.1 points) (*p*=0.02) (Figure 2). Another secondary finding was a significant decrease by −2.0 mmHg in IOP between the baseline and 12 weeks (*p* < 0.001), which was included in the clinical parameters as a reference value to detect any clinical worsening or any influence of the HA-based formulation on the effect of PG analog therapy.

## 4. Discussion

The results of the present study support the efficacy of an ophthalmic formulation containing HA, amino acids, and *Echinacea* in the treatment of ocular symptoms associated with DED in patients undergoing treatment with PG analogs. The present study examined several outcomes, all of which may be representative of ocular surface changes. Indeed, daily use of the study ophthalmic formulation resulted in rapid improvement in inflammation-related symptoms and their frequency, with a significant decrease in burning sensation after only 4 weeks of treatment; foreign body sensation, dryness, and pain scores were also statistically significantly lower after 12 weeks of treatment than at the baseline. The improvement in these symptoms is crucial, since lower levels of inflammation help break or at least mitigate the typical vicious cycle of DED, in which inflammation is not only caused by the ocular surface but also becomes a key factor in damage to the eye [31]. These positive outcomes were confirmed by the self-completed OSDI questionnaire, in which scores improved significantly after both 4 and 12 weeks of treatment. Changes in objective parameters, such as tear stability (TBUT test), which is severely impaired in DED patients and is one of the concomitant phenomena leading to ocular surface stress,also mirrored the clinical results; conjunctival hyperaemia and basal tear secretion (Schirmer test) data reinforced the observed improvement in questionnaire scores, although not with statistically significant differences. Considering the chronic nature of the disease, it is important to highlight that most of the effects promoted by the HA-based ophthalmic formulation in this study were visible after 4 weeks of treatment and persisted throughout the study period. These effects can be attributed to the distinctive composition of the ophthalmic formulation studied, as similar results have been observed in several previous clinical studies with HA-based eye drops [32–34]. Indeed, Molina-Solana et al. conducted a prospective, single-arm longitudinal intervention study to evaluate the efficacy of a preservative-free artificial tear containing 0.4% HA and found a significant improvement in signs and symptoms, such as hyperaemia, CFS, and OSDI score, after 1 week and 1 month of treatment [33]. Similar results were also obtained in the study by Sanchez-Gonzalez et al., who recorded an improvement in Schirmer test results, TBUT, and OSDI score after artificial tears containing different concentrations of HA were administered [34]. Among others, Roberti et al. conducted a prospective, randomized, single-masked, parallel study to evaluate the efficacy of a preservative-free solution containing 0.4% HA and 0.5% taurine in glaucoma patients undergoing long-term treatment with preserved hypotensive therapy. Their results showed that the formulation greatly improved the signs and symptoms associated with DED [35]. Finally, the safety and efficacy of HA-based artificial tears were thoroughly investigated by Aragona et al. in a randomized, controlled, multicenter, 3-month study involving >460 patients [36]. An interesting point that emerged from this study is the possible synergistic effect of formulation components. Indeed, the formulation tested here was highly effective compared to those investigated in the aforementioned studies despite the lower concentration of HA [28]. Thus, such an effect could be due to the combination of HA with amino acids. Indeed, supplementation with amino acids, especially L-proline, L-lysine, L-glycine, and L-leucine, is known to support the metabolism of the corneal epithelium, which is damaged in DED patients [37]. In the present study, the effect of treatment with the HA-based study formulation on the patients' quality of life was also evaluated using the NEI-VFQ-25 questionnaire. The results showed a significant improvement in the ocular pain score, further extending the clinical effectiveness of preparation. Although this domain was the only one in the questionnaire that showed a statistically significant improvement, the result was clinically relevant as pain is the main reason for the impairment in quality of life documented in DED patients [38]. However, these results are still preliminary due to the limited sample of the study, and a future, more in-depth analysis of quality of life involving different types of questionnaires would be of great interest. Interestingly, a statistically significant decrease in IOP was observed in patients. It seems that improving ocular surface health in glaucoma patients allows for a better control of IOP values. However, the change in IOP could be due to (i) better adherence to glaucoma treatment, which is common in patients participating in a scientific study, (ii) better adherence to glaucoma treatment due to the patient's perceived improvement in symptoms of ocular discomfort, or (iii) treatment of the ocular surface disease that allows to reduce inflammation, thus improving both ocular surface health and IOP values [33]. In future studies, it would be useful to extend the follow-up period to assess how long the effect of the study formulation lasts. Although HA-based eyedrops have been used for many years, longer observation would allow further detection and monitoring of potential adverse effects. In addition, the presence of a control group and, as mentioned above, a larger cohort of patients would be useful to better study the effects of treatment on the patients' quality of life. We acknowledge that the presence of a placebo effect might have influenced the subjective results of our study given the lack of a control group [40]; however, this was meant to be a pilot study, and in addition, the relevant improvements in clinical signs seem to support the role of the studied supplementation in improving DED in patients treated with PG analogs.

Despite the limitations mentioned above and its open-label nature, the results of this study show that the beneficial effects of eye drops containing HA, amino acids, and *Echinacea* extract are rapid and persist throughout the treatment period (12 weeks). Future randomized controlled trials would be necessary to better define the application regimen and extend the use of the ophthalmic formulation to other ocular conditions.

## Figures and Tables

**Table 1:** Patients' characteristics at the baseline.

Characteristics	Level	Statistics^a^
Sex	Male	15 (50.0)
	Female	15 (50.0)

Age (years)	Male	70.1 (10.8)
	Female	64.2 (10.5)
	Overall	67.1 (10.8)^b,c^

Duration of glaucoma (years)		9.1 (5.0–12.0)

Duration of therapy (years)	Actual	6.0 (3.0–10.0)
	Total	8.3 (4.0–12.0)

^a^Statistics are displayed as count (%) for sex or mean (standard deviation) for age but mean (interquartile range) otherwise. ^b^Min = 44.4, max = 87.0. ^c^Male vs. female unpaired *t*-test, *p*=0.14.

**Table 2:** Summary statistics for secondary outcomes by time.

Outcome	Visit time	*N*	Mean (standard deviation)		*p* value^a^
			Score	Paired difference with baseline	
OSDI	Baseline	30	37.7 (18.1)		
	4 weeks	30	29.9 (17.8)	−7.8 (16.7)	**0.02**
	12 weeks	30	29.6 (18.8)	−8.1 (20.7)	**0.04**

TBUT (seconds)	Baseline	30	4.7 (3.1)		
	4 weeks	30	6.1 (3.8)	1.5 (3.6)	**0.01**
	12 weeks	30	4.9 (1.9)	0.2 (2.5)	0.40

CFS	Baseline	30	5.4 (3.6)		
	4 weeks	30	3.3 (2.4)	−2.1 (2.8)	**<0.001**
	12 weeks	30	0.9 (1.4)	−4.5 (4.0)	**<0.001**

Schirmer test (mm)	Baseline	30	13.6 (7.4)		
	12 weeks	30	15.7 (5.1)	2.1 (5.7)	0.05

^a^One-sample signed rank-sum test or 1-sample*t*-test as appropriate. Abbreviations: CFS, corneal fluorescein staining; OSDI, Ocular Surface Disease Index; TBUT, tear film break-up time.

## Data Availability

The datasets generated during and/or analysed during the current study are available from the corresponding author on reasonable request.
